# Self-administration medication errors at home and its predictors among illiterate and low-literate community-dwelling older adults with polypharmacy: A negative binomial hierarchical regression

**DOI:** 10.1371/journal.pone.0302177

**Published:** 2024-04-19

**Authors:** Nafiseh Ghassab-Abdollahi, Haidar Nadrian, Elnaz Shaseb, Narges Kheirollahi, Mina Hashemiparast

**Affiliations:** 1 Department of Geriatric Health, Faculty of Health Science, Tabriz University of Medical Sciences, Tabriz, Iran; 2 Department of Health Education & Promotion, School of Health, Tabriz University of Medical Sciences, Tabriz, Iran; 3 Department of Pharmacotherapy, Faculty of Pharmacy, Tabriz University of Medical Science, Tabriz, Iran; 4 Social Determinants of Health Research Center, Zanjan University of Medical Sciences, Zanjan, Iran; 5 Department of Health Education & Promotion, School of Public Health, Zanjan University of Medical Sciences, Zanjan, Iran; Khalifa University, UNITED ARAB EMIRATES

## Abstract

**Background:**

Older adults with polypharmacy are more prone to medication errors. People with low educational attainment have more difficulties in taking their medications.

**Objectives:**

This study aimed to identify the extent of medication self-administration errors (MSEs) and the contributing factors among illiterate and low-literate community-dwelling older adults with polypharmacy.

**Method:**

The present cross-sectional study was conducted among people aged 60 and above. The data were collected using the sociodemographic, clinical, and Belief about Medicines Questionnaires (BMQ). To determine the extent of MSE, a medication error checklist was used. The negative binomial hierarchical regression model in the five blocks was performed.

**Results:**

The final sample size was 276 people. The frequency of MSEs in the last 6 months was 69.2%. Sixteen percent of participants had made four or more mistakes. The most common MSEs were forgetting, improper taking of medications with food, improper timing, incorrect dosage (lower dose), and forgetting the doctor’s instructions. Near 18% of participants reported adverse events following their mistakes. The significant predictors of MSEs were being completely illiterate (p = 0.021), the higher number of doctor visits per year (p = 0.014), irregularly seeing doctors (p < .001), the higher number of medications (p < .001), and having poor medication beliefs (p < .001).

**Conclusion:**

Despite the high prevalence of MSEs among older patients, practical strategies to deal with them at their homes have not been established among health systems. MSE as a multifactorial event can be caused by a collection of internal and external factors. Further studies to identify the role of patients, clinicians, procedures, and systems in developing MSEs as interconnected components are needed.

## Introduction

The National Coordinating Council for Medication Error defines a medication error as “any preventable event that may cause or lead to inappropriate medication use”[1]. According to this definition, medication errors may occur at any stage, including prescribing, compounding, dispensing, distribution, and administration [2]. Previous studies have mainly focused on medication errors caused by health professionals [3–5]. However, in many cases, patients themselves commit medication errors during self-administration [6]. A medication self-administration error (MSE) at home occurs when a medication is prescribed by the physician but taken at the wrong time or dose, confused with other medications, or wrongly stored [7].

Medication errors are a major concern for health systems because they reduce treatment effectiveness and increase costs. Medication errors often have no negative consequences for a patient but, in other cases, may threaten patient safety. These errors may expose the patients to more severe consequences, such as hospitalization or death [8].

The older population often suffers multimorbidity, which results in receiving multiple medications [9]. Polypharmacy, defined as the regular use of at least five medications, is highly prevalent among older adults compared to other age groups. Polypharmacy has been reported in almost half of patients aged 65 and older. As a result, people with polypharmacy are more prone to medication errors [10, 11]. MSEs frequently occur in older individuals, especially among those who take more than five medications [2].. In Spain, the frequency of medication errors among older patients with multimorbidity was reported at 75% [12].

Older people with limited education are more exposed to medication errors. Low literacy and multiple medications are associated with misunderstanding medication instructions. Patients with low literacy skills demonstrated a higher rate of medication label misinterpretation [13]. People with low educational attainment have more difficulty in reading and understanding written information and ranges of values. They tend to have poorer baseline knowledge compared to people with higher literacy [14]. Therefore, the rates of MSEs and the variety of the most common MSEs in low-literate older adults are expected to be different.

There are some previous limited studies about the contributing factors of MSEs in older adults. Polypharmacy and reduced cognitive and physical abilities have been reported as predictors of medication errors [2]. More information is needed to clarify and confirm the results among independent community-dwelling older adults with polypharmacy at their homes without limiting them to a particular disease. Moreover, studies on medication use in older adults have mainly focused on the predictors of medication adherence, and few studies examine the predictors of MSEs [7].

Although most errors occur in the community setting in older adults’ homes, there are few studies in this regard [2]. Although limited formal education is still a common problem among older populations, especially in developing countries [15], limited studies evaluate all types of possible self-administration errors among low-literate older adults. Recognizing factors relating to MSEs can orient healthcare professionals in clinical practice and help them to provide the necessary supportive care and to design effective interventions [2]. So, based on previous studies [7, 12], we hypothesized that the older adults’ profile, medical and medication history, and beliefs about medication and receiving support can affect MSEs. Therefore, this study aimed to identify the extent of MSEs and their contributing factors (including sociodemographic, medical history, medication-related factors, medication belief, and monitoring of medications by a caregiver) among illiterate and low-literate community-dwelling older adults who take at least 5 medications in their homes via hierarchical regression.

## Materials and methods

### Study design and population

The present cross-sectional study was conducted among illiterate and low-literate (Five years and less) community-dwelling older adults aged 60 years and above who were taking five or more medications in the city of Tabriz-Iran. Patients who had been diagnosed with dementia or those with an Abbreviated Mental Test score (AMT) of less than eight were excluded from the study. Older adults who lived in nursing homes and other care centers, and were hospitalized, physically dependent, immobile, or homebound were also excluded from the study. The Strengthening the Reporting of Observational Studies in Epidemiology (STROBE) Statement was used to report data [16].

G*Power (version 3.1.2) software (Franz Faul, Universitat Kiel, Germany) was used to determine the sample size. Based on the study of Mira et al., [12] the frequency of medication errors among older poly-medicated patients was estimated at 75%. Considering α = 0.05, 95% confidence interval, and 10% loss, the sample size was calculated as 318 people. Forty-two participants (15%) refused replay. So, 276 patients completed the study.

### Sampling

**Fig 1:**
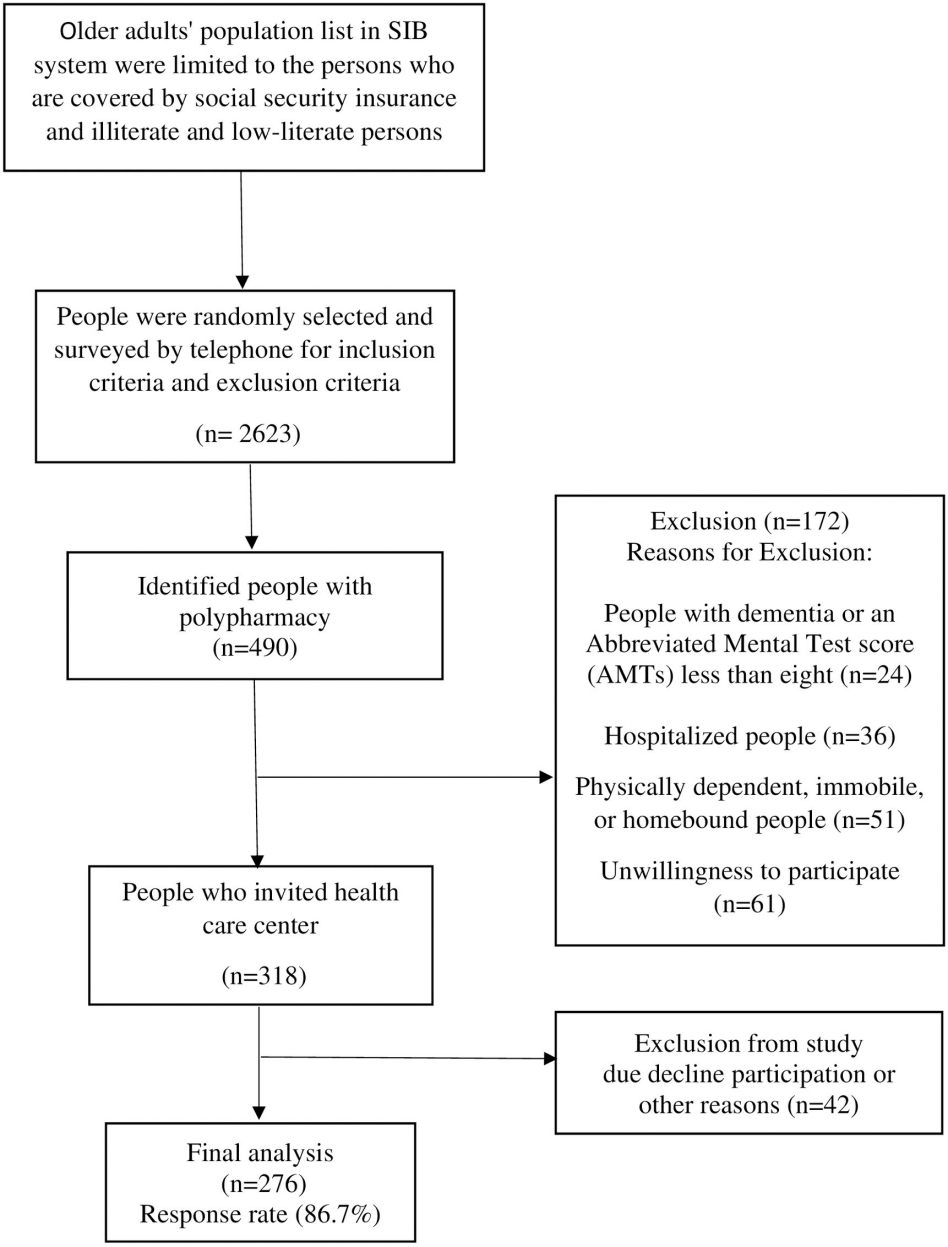
Flow chart for study subject selection.

The sampling procedure was carried out in Tabriz health centers from March to August 2022. The city of Tabriz has 87 health centers, and the information on all older adult residents is available through The Integrated Health System (SIB). A list of all people aged 60 and above along with their telephone numbers was obtained from SIB. Since, only the Social Security Organization (SSO), and a few other insurance organizations store the information of patients’ prescriptions electronically, in this study the participants were limited to the persons who are covered by social security insurance. SSO as the oldest insurance organization in Iran has the highest rate of insurance coverage approximately more than 60%. The electronic registration system of SSO provides access to the electronic prescriptions of all covered patients. The SIB system allows users to limit the population to illiterate and low-literate persons and insurance kind by data filtering. After limiting the population to illiterate and low-educated people with social security insurance, 318 people were randomly selected by a simple random method, using online software (www.random.org). Then, the researcher contacted the selected people and assessed the inclusion and exclusion criteria. Eligible people were invited to attend the health center and bring all their medications. In a face-to-face meeting, the researcher explained the aims of the study. Detailed information on the recruitment of study participants is provided in Fig 1. After the enrollment, the questionnaires were completed by the researcher.

### Measurements

The data collection instruments consisted of a six-part researcher-made questionnaire including sociodemographic, medical, and medication-related information, a caregiver profile questionnaire, a medication error checklist, and the consequences of MSEs. A medication belief questionnaire as a standard tool was also used.

*The sociodemographic questionnaire* was used to obtain information on age, gender, education (illiterate vs. low-literate (Five years of elementary education and less)), marital status, number of children, employment status, living arrangements, family income, and smoking.

*Medical history* consisted of questions about the number of chronic diseases, hearing and vision status, BMI (kg/ m^2^), number of physicians, doctor visits per year, and doctor checkup status.

*Medication-related information* gathered information on the number of medications, months of treatment with five or more drugs, medication refill, treatment satisfaction, side effects of medications, and support tools used at home.

*Caregiver support questionnaire*: in this questionnaire, the participants were asked if anyone helped them with their medications. If the participant received help from anyone, the caregiver profile was obtained.

*Belief about Medicines Questionnaire (BMQ)* [17] contains two five-item scales: necessity and concerns. The necessity part assesses patients’ beliefs about their personal need for the prescribed medicine, while the part of the concern assesses concerns about the medications. Each question is scored on a 5-point Likert scale (1 = completely disagree, 2 = disagree, 3 = neutral, 4 = agree, 5 = completely agree). The total scores for the necessity and concern parts are between 5 and 25. A necessity–concerns differential is calculated, with a possible range of -20 to +20. Higher scores demonstrate stronger beliefs. The higher scores indicate the patients’ belief in the usefulness of their medication [18]. BMQ was translated into the Persian language by Minaiyan et al. and the reliability of questionnaire has been confirmed [19].

*Medication error checklist* in this study, a checklist developed by the research team was used to investigate common medication errors among older adults in the last six months. Using a literature review, the most common possible medication errors in self-administration were extracted from previous studies [2, 6, 7, 12, 20–22]. The details of checklist items and examples of error types are listed in the S1 File. The researcher asked the participants to state the dose, time, and route of taking their medications. The participants’ answers were compared with their electronic medical records. Any unintentional discrepancy between the electronic medical record and the participant’s answers was considered a medication error. Other MSEs such as forgetting to take medications and rout of medication storage at home were checked by patient self-reports. This method of medication error assessment was designed based on the studies conducted by Mira et al. (2013) and Mira et al. (2014). We also asked participants to demonstrate how to use devices (such as inhalers, and insulin pens). To increase the accuracy of the information, the participants were asked to be accompanied by one of their family members or relatives. In the present study, we considered only errors that occurred unintentionally by the participants, for example, intentional changes or omissions of medication dosage due to non-compliance with treatment were not considered as MSEs. A pharmacist (Shaseb, E) reviewed the participants’ medications and compared the answers. Previous studies considered one year [12, 20], but in this study, we limited this period to the last six months to reduce recall bias. In this study, the chronic use of medications was considered (The duration of medication use ≥30 days).

*The consequences of the medication error questionnaire* included questions about whether a medication error had caused an adverse outcome in the participant.

The validity of researcher-made questionnaires and checklist was determined using face and qualitative content validity. The research team including a pharmacist evaluated the level of difficulty, suitability, and ambiguity of the questionnaires. The questionnaires were sent to 10 expert faculty members and asked them to submit their comments about the content of the items, grammar, wording, and the overall format of the questionnaires. The necessary changes were made based on their comments. Before sampling, the researcher completed the questionnaires for ten illiterate older adults with polypharmacy. The necessary changes and corrections were given based on their answers to the questionnaires.

### Ethical considerations

This study was approved by the Research Ethics Committee of Tabriz University of Medical Sciences (code: IR.TBZMED.REC.1400.974). The research team prepared the written informed consent. Because of the participants’ poor reading skills, before obtaining informed consent from them, the researcher read the contents of the form in plain language and asked them to explain the received meaning from the statements. The subjects were assured about the confidentiality of their information and voluntary participation in this study.

### Statistical analysis

SPSS software (version 24) was used for data analysis. Descriptive statistics including frequency, percentage, mean and standard deviation (SD) were used to describe the MSE rate and sociodemographic and medical, and medicinal characteristics. To determine factors contributing to MSEs the negative binomial hierarchical regression model was performed. The variables were categorized into five blocks: sociodemographic factors (block 1), medical history (block 2), medication-related factors (block 3), medication belief (block 4), and caregiver support (block 5). The selection of variables within the blocks was done by backward strategy and by removing variables with p-value < 0.1. The selected variables of each block were added step by step to the important variables of the previous blocks. In all statistical analyses, P < 0.05 was considered significant.

## Results

### Participant characteristics

The final sample size was 276 people. The average response rate was 86.7%. The flow diagram of participant selection is shown in Fig 1. The results showed that the average age of the participants was 69.55 ± 6.1 years. More than half of them were female (58.3%), housewives (54.7%), low-educated (64.1%), married (81.2%), and living with their spouses (81.2%). The average number of children for each participant was 4.04 ± 1.9. The Income of thirty-eight percent of the participants was relatively sufficient. Smoking was reported by 7% of them (Table 1).

**Table 1:** Participant characteristics (n = 276).

Variables		n	%
Age /Mean (SD)	Year	69.55 (6.1)	
Gender	Male	115	41.7%
	Female	161	58.3%
Job	Housewife	151	54.7%
	Employed	31	11.2%
	Retired	80	29.0%
	Retired and employed	14	5.1%
Education	Illiterate	99	35.9%
	Low-literate	177	64.1%
Marital status	Married	224	81.2%
	Widow/Divorced/Single	52	18.8%
Living order	Alone	31	11.2%
	With spouse	224	81.2%
	With or children or other relatives	21	7.6%
Number of children/Mean (SD)		4.04 (1.9)	
Income status	Sufficient	94	34.1%
	Somehow sufficient	104	37.7%
	Insufficient	76	27.5%
Smoking	Yes	19	6.9%
	No	257	93.1%
Number of chronic diseases	1	15	5.4%
	2	61	22.1%
	3	108	39.1%
	≥4	92	33.3%
Visual status	Normal	92	33.3%
	Mild impairment	176	63.8%
	Severe impairment	8	2.9%
Auditory status	Normal	219	79.3%
	Mild impairment	37	13.4%
	Severe impairment	20	7.2%
BMI/Mean (SD)	kg/m^2^	29.51 (4.4)	
Number of physicians	Without a physician or without a constant physician	40	14.5%
	1	111	40.2%
	2	71	25.7%
	3	21	7.6%
	4≥	10	3.6%
Doctor visits per year/Mean (SD)		3.73 (2.8)	
Regular doctor checkup	Yes	182	66.4%
	No	92	33.6%
Number of medications	5	64	23.2%
	6–8	127	46.0%
	9≥	85	30.8%
Duration of medication takin /mean (SD)	Month	95.86 (79.2)	
Medication satisfaction	Weak/Moderate	29	10.5%
	Good	76	27.5%
	Excellent	171	62.0%
Side effects of medications	Yes	30	10.9%
	No	246	89.1%
Medications refills after last prescription per year/Mean (SD)		3.44 (3.1)	
Support tools used at home	None	243	88.4%
	Pillbox	32	11.6%
	Calendar	1	0.4%
Do you have someone who helps you with your medications? (Having an informal caregiver)	Yes	145	52.5%
	No	131	47.5%
Gender of caregiver	Male	50	34.7%
	Female	94	65.3%
Relationship of caregiver	Spouse	22	15.5%
	Children	100	70.4%
	Other	20	14.1%
Caregiver Education	Illiterate and primary education	21	14.6%
	Secondary and high school	22	15.3%
	Diploma	50	34.7%
	Associate’s degree and College education	51	35.4%
Medication belief/Mean (SD)	Necessity (5 to 25)	18.84 (5.2)	
	Concern (5 to 25)	10.38 (4.8)	
	Total (-20 to +20)	8.45 (7.3)	

SD: Standard Deviation, Low-literate: Five years of elementary education and less, Necessity: the necessity part assesses patients’ beliefs about their personal need for the prescribed medicine, Concern: the part of the concern assesses concerns about the medications, Total: A necessity–concerns differential is calculated as the difference between the necessity and the concerns parts, with a possible range of -20 to +20.

As shown in Table 1, 39.1% of the participants were suffering from three chronic diseases. More than half of the older adults reported mild visual impairment (63.8%). Most of the participants had normal hearing status (79.3%). About 40% of the participants had one physician and the average annual number of visits was 3.73 ± 2.8. Almost 66% of older adults saw their doctor regularly.

Forty-six percent of the participants were chronically taking 6 to 8 medications, and the average duration of receiving the medication was 95.86 ± 3.1 months. Sixty-two percent of participants reported excellent satisfaction with their medication regimen, and near to 11% of them reported medication side effects. On average, participants refilled their medications 3.44 ± 3.1 times after their last prescription. The majority of older adults (88.4%) did not use any support tools at home (Table 1).

More than half of the participants (52.5%) had someone (informal caregiver) to help them with their medications. Most of the caregivers were female (65.3%) and 70.4% of them were children of older adults. Approximately 35% of participants had an associate’s degree or college education. The results showed that the mean necessity score of BMQ was 18.84 ± 5.2 and the mean concerns score was 10.38 ± 4.8. The average total score of BMQ was calculated at 8.45 ± 7.3 (Table 1).

### Frequency of MSEs

The results showed that the frequency of MSEs among illiterate and low-literate older adults in the last 6 months was 69.2%. Near 31% of participants had not committed any error. It was detected that 16.3% of participants had made four or more mistakes with their medications during the past 6 months (Table 2).

**Table 2:** The frequency of self-administration errors among illiterate and low-literate older adults with polypharmacy in the last 6 months (n = 276).

	N	%
The total frequency of error	191	69.2%
Without error	85	30.8%
1 Error	77	27.9%
2 Errors	46	16.7%
3 Errors	23	8.3%
4 Errors and more	45	16.3%

The results demonstrated that the most common MSE among older adults was forgetting to take medications (37.3%). Subsequently, six common MSEs in the past 6 months were as follows: improper taking of medications with food, beverages, and herbs (17.8%), improper timing (13.8%), incorrect dosage (lower dose) (13.0%), forgetting the doctor´s or pharmacist’s instructions (13.0%), incorrect dosage (higher dose) (12.7%) and taking expired medications (10.9%) (Table 3).

**Table 3:** The frequency of self-administration medication errors by different types of error among illiterate and low-literate older adults with polypharmacy in the last 6 months (n = 276).

Types of self-administration medication errors		N	%
*Forgetfulness*		103	37.3%
*Improper timing*		37	13.8%
*Incorrect dosage*	*Higher dose*	35	12.7%
	*Lower dose*	36	13.0%
	*Duplication*	13	4.7%
*Taking the wrong medication because of similar appearance (Look-alike packaging)*		18	6.5%
*Incorrect route of administration*		11	4.0%
*Improper taking of medications with food*, *beverages*, *and herbs*		49	17.8%
*Improper taking of medications together*		2	0.7%
*Medication omission*		5	1.8%
*Taking previous medications*		20	7.2%
*Taking another person’s medications*		1	0.4%
*Errors related to medication delivery involve devices*		8	2.9%
*Improper Medication Storage*		25	9.1%
*Taking expired medications*		30	10.9%
*Self-medication (Taking medications without a prescription)*		25	9.1%
*Forgetting the doctor*’*s or pharmacist*’*s instructions*		36	13.0%
*Mistaken drug because of misunderstanding of the purpose of the medication*		13	4.7%

### Consequences of MSEs

As shown in Table 4, among participants who had made mistakes in the last six months, 17.8% reported adverse events following their mistakes. More than half of them (61.8%) reported that the adverse event was mild. Only 2 participants (5.9%) reported severe adverse events. Out of the patients who reported adverse events, 3 patients (8.8%) needed intervention.

**Table 4:** The consequences of medication errors among participants who had committed an error (n = 191).

		N	%
Adverse events of medication errors	Yes	34	17.8%
	No	157	82.1%
Severity of adverse events	Mild	21	61.8%
	Moderate	11	32.4%
	Severe	2	5.9%
Need to intervention	Yes	3	8.8%
	No	31	91.2%

Predictors of MSEs among illiterate and low-literate community-dwelling older adults

To determine the predictors of MSEs, we used the negative binomial hierarchical regression. Among all variables of the study, the variables with p < 0.1 were selected by the backward variable selection method and hierarchically entered into the model. Table 5 presents the summary of hierarchical regression. The results showed that in the first model, illiterate people commit mistakes in their medications 1.62 times more than low-literate older adults (OR 1.62, 95% CI: 1.19–2.20; p = 0.001). In the second model after including the participants’ medical history, the results indicated that being illiterate (OR 1.44, 95% CI: 1.05–1.98; p = 0.021), the higher number of doctor visits per year (OR 1.08, 95% CI: 1.01–1.15; p = 0.014) and having irregular doctor checkups (OR 0.44, 95% CI: 0.29–0.65; p < .001) significantly related to the higher number of MSEs in the last 6 months. By adding medication-related factors in the third model, the analysis again showed that having regular doctor checkups (OR 0.45, 95% CI: 0.29–0.68; p < .001) significantly reduces MSEs. Polypharmacy is significantly related to MSEs. The results also indicated that the participants who take 5 medications (OR 0.42, 95% CI: 0.26–0.67; p < .001) have a lower MSE than those who take 9 medications or more. After incorporating medication belief in model 4, the analysis showed that irregularly seeing a doctor, the higher number of medications, and poor medication belief are significant predictors of MSEs. For every one-unit increase in the total score of medication belief, the MSEs decrease by 0.95 (OR 0.95, 95% CI: 0.93–0.98; p < .001). In the final model after adding having a caregiver, irregularly seeing a doctor (OR 0.41, 95% CI: 0.27–0.62; p < .001), the higher number of medications (OR 0.40, 95% CI: 0.24–0.64; p < .001), and lower medication belief (OR 0.95, 95% CI: 0.93–0.98; p < .001) were significant predictors of MSEs.

**Table 5:** Summary of hierarchical regression analysis for variables predicting medication adherence.

		B	S.E.	Wald *X*^2^	P-value	OR (95% CI)
**Model** 1						
*Education*	Illiterate	0.483	0.15	9.59	0.001	1.62 (1.19–2.20)
	Low-literate	0	-	-	-	-
**Model 2**						
*Education*	Illiterate	0.37	0.16	5.28	0.021	1.44 (1.05–1.98)
	Low-literate	0	-	-	-	-
*Number of doctor visits per year*		0.07	0.03	5.99	0.014	1.08 (1.01–1.15)
*Regular doctor checkup*	Yes	-0.81	0.20	16.35	< .001	0.44 (0.29–0.65)
	No	0	-	-	-	-
**Model 3**						
*Education*	Illiterate	0.24	0.16	2.09	0.148	1.27 (0.91–1.77)
	Low-literate	0	-	-	-	-
*Number of doctor visits per year*		0.05	0.03	3.08	0.079	1.06 (0.99–1.13)
*Regular doctor checkup*	Yes	-0.79	0.21	14.22	< .001	0.45 (0.29–0.68)
	No	0	-	-	-	-
*Number of medications*	5	-0.85	0.23	13.11	< .001	0.42 (0.26–0.67)
	6–8	-0.34	0.18	3.53	0.060	0.71 (0.49–1.01)
	9≥	0	-	-	-	-
*Duration of medication taking*	Month	-0.002	0.001	2.83	0.092	0.99 (0.99–1.0)
*Medication satisfaction*	Weak/Moderate	0.43	0.25	2.94	0.086	1.54 (0.94–2.52)
	Good	0.11	0.18	0.36	0.545	1.12 (0.77–1.61)
	Excellent	0	-	-	-	-
*Side effects of medications*	Yes	0.42	0.24	2.99	0.084	1.52 (0.94–2.44)
	No	0	-	-	-	-
**Model 4**						
*Education*	Illiterate	0.23	0.17	1.81	0.179	1.25 (0.90–1.76)
	Low-literate	0	-	-	-	-
*Number of doctor visits per year*		0.05	0.03	2.99	0.084	1.06 (0.99–1.13)
*Regular doctor checkup*	Yes	-0.88	0.21	17.24	< .001	0.41 (0.27–0.62)
	No	0	-	-	-	-
*Number of medications*	5	-0.95	0.24	15.76	< .001	0.38 (0.24–0.61)
	6–8	-0.37	0.18	4.03	0.045	0.69 (0.48–0.99)
	9≥	0	-	-	-	-
*Duration of medication taking*	Month	-0.002	0.001	3.20	0.073	0.99 (0.99–1.0)
*Medication satisfaction*	Weak/Moderate	0.24	0.25	0.86	0.353	1.27 (0.76–2.11)
	Good	0.05	0.18	0.09	0.763	1.05 (0.73–1.53)
	Excellent	0	-	-	-	-
*Side effects of medications*	Yes	0.21	0.25	0.74	0.387	1.24 (0.75–2.03)
	No	0	-	-	-	-
*Medication belief*		-0.04	0.01	14.42	< .001	0.95 (0.93–0.98)
**Model 5**						
*Education*	Illiterate	0.15	0.18	0.76	0.381	1.17 (0.82–1.67)
	Low-literate	0	-	-	-	-
*Number of doctor visits per year*		0.05	0.03	2.39	0.121	1.05 (0.98–1.12)
*Regular doctor checkup*	Yes	-0.89	0.21	17.27	< .001	0.41 (0.27–0.62)
	No	0	-	-	-	-
*Number of medications*	5	-0.91	0.24	14.20	< .001	0.40 (0.24–0.64)
	6–8	-0.34	0.18	3.42	0.064	0.70 (0.49–1.02)
	9≥	0	-	-	-	-
*Duration of medication taking*	Month	-0.002	0.001	3.08	0.079	0.99 (0.99–1.0)
*Medication satisfaction*	Weak/Moderate	0.20	0.26	0.58	0.445	1.22 (0.73–2.03)
	Good	0.03	0.19	0.02	0.876	1.03 (0.70–1.49)
	Excellent	0	-	-	-	-
*Side effects of medications*	Yes	0.23	0.25	0.87	0.350	1.26 (0.77–2.07)
	No	0	-	-	-	-
*Medication belief*		-0.04	0.01	14.52	< .001	0.95 (0.93–0.98)
*Having a caregiver*	Yes	0.21	0.17	1.50	0.220	1.24 (0.87–1.76)
	No	0	-	-	-	-

All variables with P < 0.1 were entered to the model with Backward strategy, Model 1 includes education, Model 2 includes medical variables, Model 3 includes medicinal variables, Model 4 includes medication belief, Model 5 having a caregiver

## Discussion

The data obtained from illiterate and low-literate community older patients with polypharmacy showed that the frequency of MSEs in the last 6 months is high. The significant predictors of MSEs analyzed from the hierarchical regression included being completely illiterate, higher number of doctor visits per year, irregularly seeing a doctor, higher number of medications, and having low medication beliefs.

### Frequency of MSEs

About 69% of older patients in the present study made mistakes with their medications in the past 6 months. In the Mira et al. study, medication errors in older patients with multimorbidity were reported by 75% in the last year [12]. A systematic review also reported the frequency of medication error among older adults with a complex therapeutic regimen increased up to 75% [7]. The frequency of errors in the current study was higher compared to Mira’s study because the current study measured the occurrence of errors in a shorter period (6 months vs. 1 year). MSEs have been examined using different definitions and criteria. Previous studies have mainly focused on errors related to dosage and a few limited errors, but this study considered more diverse MSEs. Most efforts to introduce types of medication errors caused by the patients are provided by Prof. José Joaquín Mira [6, 12]. Despite these efforts, a comprehensive checklist of all possible types of MSEs has not been developed so far. We identified 19 types of MSEs through a comprehensive literature review. Therefore, it can be justified that the frequency of MSEs identified in the present study is higher than in previous studies.

It should be acknowledged that discovering MSEs at home is not an easy task when it is away from the observation of a clinician [23]. Previous studies have suggested further research to develop better ways of identifying MSEs [24]. We applied a combination of different methods such as matching the prescriptions with patients’ answers, looking over the medications by the researcher (e.g., checking the drug expiration dates), asking the patient to demonstrate and explain the technique of using the devices, and discovering errors with the help of a family member or relative. However, it cannot be claimed that all errors committed by the patients at their homes can be identified especially errors that are not reported due to recall bias or when patients may not realize their mistakes due to the absence of error-related adverse events.

The results showed that the most common MSE among older adults was forgetting to take medications. In a systematic review, the most common error was dosage mistakes. Other reported errors were: forgetting, taking the wrong medicine, duplication, wrong preparation methods, wrong ways of taking the medicine, wrong time, wrong frequency, and use of expired medications [2]. Low education has negative effects on the working memory of older people. In other words, illiterate seniors have less ability to keep information in their minds [25, 26]. In this study, we only included people who did not have cognitive impairment. It seems that the high percentage of forgetfulness among the participants can be partially attributed to the lower level of education. Altogether, it can be said that the error pattern among illiterate and low-educated seniors is somewhat similar to the general older population, but the error occurrence rate is higher. However, it should not be forgotten that the frequency of types of medication errors will be different depending on error evaluation checklists. On the other side, more errors are associated with more side effects for the patient. A greater awareness of the frequency of patient errors leads to a better awareness of errors’ consequences in clinical terms and also concerning patients’ quality of life and health system costs [12].

Seniors with low education levels are not able to effectively use error reduction strategies such as new technologies. The use of strategies that require fewer skills should be developed. Using wristwatch alarms, phone calls to remind medications, and reminder systems in smart homes such as turning on the lights, and turning on the TV screen can help to reduce forgetfulness. Supporting older adults in the use of these approaches by caregivers and family members is beneficial.

In this study, self-medication (taking medications without a prescription) was calculated as 9%. The results of the present study are not consistent with previous studies. The prevalence of self-medication among older adults in a systematic review has been reported as high as 36% (varied from 0.3% to 82%). Various definitions of self-medication and different populations have created such inconsistency [27]. We considered only the chronic use of medications, which could be the reason for the low percentage of self-medication in the present study.

### Consequences of MSEs

In accordance with previous reports, the results of the present study also demonstrated that the majority of errors have no negative consequences for patients. About 18% of patients who committed an error reported the occurrence of an adverse event. Among them, only three patients needed intervention such as medication therapy or hospitalization. Mira et al. study reported that no changes in biochemical parameters indicate that the MSEs did not have a significant adverse effect on the participants [20]. However, it should be noted that some errors are never detected by the patient and clinicians. So, the possible consequences related to these errors remain also hidden. On the other hand, the consequences of MSEs were evaluated only based on the patient’s perspective. Therefore, this type of assessment may introduce biases regarding the accuracy of perceived severity of consequences by patients.

### Predictors of MSEs

A previous systematic review [2] reported that MSEs in older adults arise from various causes. In addition to polypharmacy, cognitive impairment, physical abilities decline, certain beliefs and attitudes towards medication, insufficient knowledge about treatment regimens, failures in communication with the clinicians, and low education are contributing factors of MSEs. A closer look at the eleven articles in this systematic review makes it clear that they have been conducted on diverse populations with different designs, tools, and settings. Some of these articles focused on specific populations or specific diseases, for example, examining medication use in people with Parkinson’s disease [28] or evaluating the use of inhalers in people with asthma at the hospital [29]. This study attempted to take a more comprehensive view of MSEs in community-dwelling seniors.

The study recognized that being illiterate is a predictor of medication error. It seems even having an elementary education is an effective factor in preventing MSEs in older people compared to being completely illiterate. Previous studies have shown that people with lower levels of health literacy committed more errors [2]. However, the role of general literacy in MSEs has not been highlighted so far. Low-educated people have more difficulty in understanding written or spoken drug information [30]. They also have more difficulty in reading and understanding drug brochures, numerical information, and doing calculations, compared to people with high literacy [14]. Further, 88% of participants did not use any support tools such as pill boxes, calendars, or alarm reminders to manage their medications. Only nearly 12% of participants used pillboxes. In Mira’s study, 46% of older adults used a pillbox to correctly manage their medication at home [20]. Older patients use a variety of external strategies to manage their medications, such as taking notes, making checklists, using an alarm clock, and using new technologies such as smartphone applications [7, 31]. These methods help to create the organization of medications and avoid confusion [32]. But the illiterate older adults cannot use these strategies. For these reasons, the probability of errors among people with a lower educational level is concentrated. So, pharmacists should provide person-centered information according to the educational background of older patients [33]. Clinicians need to develop plain language for communication with low-literate older adults in their cultural context. Using Pictorial aids to help explain complex instructions may be a useful method to convey information to low-educated people [14]. It should be noted that engaging patients in developing culturally adapted pictograms based on their preferences and comprehension is essential [34, 35].

It seems that the higher number of doctor visits per year affects the MSE by increasing the number of medications used. Patients who consult multiple specialists and have a higher number of prescribers tend to be at risk of polypharmacy [36]. One study reported older adults with five or more chronic diseases have, 50 prescriptions filled, 14 different physicians, and make 37 physician visits per year [37]. All of this leads to a more complicated drug regimen and more confusion for patients, especially when instructions are inconsistent [6]. On the other hand, the results of the present study showed that irregular doctor checkup also increases the probability of errors. There is no previous data regarding the role of irregular visits in MSE. This can be explained by the fact that regular doctor visits can keep patients informed by keeping patient information up to date and helping them maintain a relationship with their doctor’s practice.

The results showed that the participants who took 5 medications had a lower MSE than those who took 9 medications or more. The findings supported existing knowledge from previous studies regarding polypharmacy’s effect on medication error [7, 12, 20]. It is clear that patients with polypharmacy are more likely to experience MSE. The results of this study showed that major polypharmacy (9 medications and more) worsens the MSE situation. Physicians’ knowledge regarding inappropriate medications in older adults is partly insufficient [38]. Physicians’ training regarding the best methods of prescribing in older patients and simplifying complex medication regimens reduces polypharmacy.

The results of a mixed-method study showed that the majority of older adults with polypharmacy believed in the necessity of their medications [39]. It seems the patients’ awareness of the cause and importance of the prescribed medications affects their medication-taking behaviors [40]. The results of this study showed that older adults, who believed that their medications were necessary, made fewer errors. Patients who know what their medication is prescribed for make fewer medication errors [21]. Doctors play an important role in informing patients. Physician communication style positively affects patients’ knowledge and initial medication beliefs [41]. Physicians need to spend more time making patients active participants in medication therapy [42].

### Implications for practice and research

MSE as a multifactorial event can be caused by a collection of internal and external factors. Accordingly, there is no single solution to error prevention. Some of the causes of MSE cannot be changed easily, or at least require long-term interventions, but other factors can be overcome by providing the proper information to the patient when prescriptions are given. Therefore, it seems necessary to design more interventions with a comprehensive approach. Moreover, weakness in health systems in providing organized pharmaceutical care and failure in establishing supportive programs for safe medication use in older adults aggravates MSE. Incorporating medication safety programs into the health care system with a patient-centered approach and with the participation of community pharmacists will reduce medication errors. On the other hand, training college students in Medical Universities in terms of communication skills and patient education, especially communication with illiterate and low-literate older patients solves many problems of medication use.

## Limitations

This study is hampered by several limitations. One of the potential limitations of this study was the nature of the cross-sectional study. The investigation was limited to the last 6 months to reduce recall bias, but some self-reporting errors may not have been reported by participants due to poorer recall among illiterate seniors. The research team tried to comprehensively investigate and identify errors using different methods, however, some of the errors may not have been detected when they occurred at home and out of sight of the researcher. The included subjects were only limited to persons covered by social security insurance. Hence, the results may not fully be extrapolated to the whole population with different insurances, due to the discrepancy in their medication use behavior according to their insurance conditions. Future comparative studies are required to compare older people with different types of insurance. In the present study, the consequences of MSEs were investigated merely based on the patient’s self-reporting. Attributing an adverse outcome to an error is not an easy task, especially when clinical and laboratory investigations are not performed. It is possible that the outcomes reported by the patients themselves were due to the side effects of other medications used by older adults and not necessarily the result of the error.

The side effects of some medicines cause memory loss, confusion, drowsiness, and sleepiness which may affect the safe use of medications. It is difficult to determine how much these complications can affect the occurrence of medication errors by older adults during chronic use of medications. It was not possible for us to examine all the factors affecting medication errors. Future studies are required to investigate other factors such as fatigue and use of hypnotics on MSEs. In this study, only the patient’s role in committing medication errors was discussed, and the possible mistakes in the received prescriptions by the patients were not considered. For example, medication errors potentially related to computerized prescriber order entry such as wrong medication selection and improper data placement, or mistakes by the pharmacist in delivering the wrong medicine to the patient.

The average age of the study participants was 69.5 years. Although the study population was randomly selected from all seniors over 60 years, older people with bone-joint problems and difficulty in climbing up the stairs, which mostly were in the higher age categories, refused participation in the study. This may affect the frequency of estimated error in this study because it is expected that the probability of error increases with age. So, to increase older people’s participation in health-related activities, it is necessary to develop health centers based on the principles of age-friendly environments.

## Conclusion

This study highlights the status of medication errors among older adults in their homes, with particular attention to illiterate and low-literate seniors. The high frequency of MSEs threatens safe medication use in older community dwellers. In the meantime, the low level of education increases the probability of making a mistake. Since seniors with low education cannot optimally use error reduction strategies such as medication management tools and new technologies, there is a need to develop simpler solutions that consider patients with limited education. Training health professionals to develop patient-centered communication skills, especially regarding older patients, to a great extent, clears up patients’ misunderstandings of medication instructions. Involving older adults and their family members in developing error prevention strategies creates more practical interventions based on patients’ preferences to prevent future errors. Further studies for a deeper understanding of the role of all the people and procedures such as patient, clinician, and system involved in causing the error as interconnected components are needed. Although programs such as home medication review can be an effective way to reduce MSEs, such programs are not common in developing countries. Despite the high prevalence of MSEs among older patients, practical strategies to deal with them have not been established among health systems and clinicians to reduce errors within the settings of seniors’ own homes.

## Supporting information

S1 File Medication self-administration errors checklist. (DOCX)

## References

[1] Food and Drug Administration. Working to Reduce Medication Errors. 2019 [cited 3 Jan 2024]. https://www.fda.gov/drugs/information-consumers-and-patients-drugs/working-reduce-medication-errors#:~:text=A medication error is defined,Medication Error Reporting and Prevention.

[2] Aldila F, Walpola RL. Medicine self-administration errors in the older adult population: A systematic review. Res Soc Adm Pharm. 2021;17: 1877–1886. doi: 10.1016/j.sapharm.2021.03.008 33811011

[3] Bakker M, Johnson ME, Corre L, Mill DN, Li X, Woodman RJ, et al. Identifying rates and risk factors for medication errors during hospitalization in the Australian Parkinson’s disease population: A 3-year, multi-center study. PLoS One. 2022;17. doi: 10.1371/journal.pone.0267969 35507635 PMC9067649

[4] Ghasemi F, Babamiri M, Pashootan Z. A comprehensive method for the quantification of medication error probability based on fuzzy SLIM. PLoS One. 2022;17. doi: 10.1371/journal.pone.0264303 35213625 PMC8880918

[5] Assunção-Costa L, de Sousa IC, de Oliveira MRA, Pinto CR, Machado JFF, Valli CG, et al. Drug administration errors in Latin America: A systematic review. PLoS One. 2022;17. doi: 10.1371/journal.pone.0272123 35925985 PMC9352042

[6] Mira JJ. Medication errors in the older people population. Expert Rev Clin Pharmacol. 2019;12: 491–494. doi: 10.1080/17512433.2019.1615442 31063401

[7] Mira JJ, Lorenzo S, Guilabert M, Navarro I, Pérez-Jover V. A systematic review of patient medication error on self-administering medication at home. Expert Opin Drug Saf. 2015;14: 815–838. doi: 10.1517/14740338.2015.1026326 25774444

[8] Pérez-Jover V, Mira JJ, Carratala-Munuera C, Gil-Guillen VF, Basora J, López-Pineda A, et al. Inappropriate use of medication by elderly, polymedicated, or multipathological patients with chronic diseases. Int J Environ Res Public Health. 2018;15. doi: 10.3390/ijerph15020310 29439425 PMC5858379

[9] Nobili A, Garattini S, Mannucci PM. Multiple Diseases and Polypharmacy in the Elderly: Challenges for the Internist of the Third Millennium. J Comorbidity. 2011;1: 28–44. doi: 10.15256/joc.2011.1.4 29090134 PMC5556419

[10] Masnoon N, Shakib S, Kalisch-Ellett L, Caughey GE. What is polypharmacy? A systematic review of definitions. BMC Geriatr. 2017;17: 1–10. doi: 10.1186/s12877-017-0621-2 29017448 PMC5635569

[11] Salazar JA, Poon I, Nair M. Clinical consequences of polypharmacy in elderly: Expect the unexpected, think the unthinkable. Expert Opin Drug Saf. 2007;6: 695–704. doi: 10.1517/14740338.6.6.695 17967158

[12] Mira JJ, Orozco-beltrán D, Pérez-jover V. Physician patient communication failure facilitates medication errors in older polymedicated patients with multiple comorbidities. Fam Pract. 2013;30: 56–63. doi: 10.1093/fampra/cms046 22904014

[13] Wolf MS, Davis TC, Tilson HH, Bass PF, Parker RM. Misunderstanding of prescription drug warning labels among patients with low literacy. Am J Heal Pharm. 2006;63: 1048–1055. doi: 10.2146/ajhp050469 16709891

[14] Baker DW, Dewalt DA, Schillinger D, Hawk V, Ruo B, Bibbins-Domingo K, et al. Teach to goal: Theory and design principles of an intervention to improve heart failure self-management skills of patients with low health literacy. J Health Commun. 2011;16: 73–88. doi: 10.1080/10810730.2011.604379 21951244 PMC3454452

[15] Pilger C, Menon MH, Mathias de TA F. Socio-demographic and health characteristics of elderly individuals: support for health services. Rev Lat Am Enfermagem. 2011;19: 1230–1238. doi: 10.1590/s0104-11692011000500022 22030589

[16] Von Elm E, Altman DG, Egger M, Pocock SJ, Gøtzsche PC, Vandenbroucke JP. The Strengthening the Reporting of Observational Studies in Epidemiology (STROBE) statement: Guidelines for reporting observational studies. Ann Intern Med. 2007;147: 573–577. doi: 10.7326/0003-4819-147-8-200710160-00010 17938396

[17] Horne R, Weinman J, Hankins M. The beliefs about medicines questionnaire: The development and evaluation of a new method for assessing the cognitive representation of medication. Psychol Heal. 1999;14: 1–24. doi: 10.1080/08870449908407311

[18] Neame R, Hammond A. Beliefs about medications: A questionnaire survey of people with rheumatoid arthritis. Rheumatology. 2005;44: 762–767. doi: 10.1093/rheumatology/keh587 15741193

[19] Minaiyan M, Taheri M, Mirmoghtadaee P, Marasi M. Comparative role of demographic factors and patient’s belief about prescribed medicine on adherence to drug treatment in chronic diseases.(in Persian). J Isfahan Med Sch. 2011;29: 1303–1311.

[20] Mira JJ, Martínez-Jimeno L, Orozco-Beltrán D, Iglesias-Alonso F, Lorenzo S, Nuño R, et al. What older complex chronic patients need to know about their everyday medication for safe drug use. Expert Opin Drug Saf. 2014;13: 713–21. doi: 10.1517/14740338.2014.916272 24821193

[21] Schwartz D, Wang M, Zeitz L, Goss ME. Medication errors made by elderly, chronically ill patients. Am J Public Health. 1962;52: 2018–2029. doi: 10.2105/ajph.52.12.2018 13987359 PMC1523132

[22] Ferner RE, Aronson JK. Clarification of terminology in medication errors: Definitions and classification. Drug Saf. 2006;29: 1011–1022. doi: 10.2165/00002018-200629110-00001 17061907

[23] Zhao M, Hoti K, Wang H, Raghu A, Katabi D. Assessment of medication self-administration using artificial intelligence. Nat Med. 2021;27: 727–735. doi: 10.1038/s41591-021-01273-1 33737750

[24] Maher RL, Hajjar ER. Medication errors in the ambulatory elderly. Aging health. 2012;8: 127–135. doi: 10.2217/ahe.12.12

[25] Roman SP. Illiteracy and older adults: Individual and societal implications. Educ Gerontol. 2004;30: 79–93. doi: 10.1080/03601270490266257

[26] Pliatsikas C, Veríssimo J, Babcock L, Pullman MY, Glei DA, Weinstein M, et al. Working memory in older adults declines with age, but is modulated by sex and education. Q J Exp Psychol. 2019;72: 1308–1327. doi: 10.1177/1747021818791994 30012055

[27] Pazan F, Wehling M. Polypharmacy in older adults: a narrative review of definitions, epidemiology and consequences. Eur Geriatr Med. 2021;12: 443–452. doi: 10.1007/s41999-021-00479-3 33694123 PMC8149355

[28] Oad MA, Miles A, Lee A, Lambie A. Medicine Administration in People with Parkinson’s Disease in New Zealand: An Interprofessional, Stakeholder-Driven Online Survey. Dysphagia. 2019;34: 119–128. Available: 10.1007/s00455-018-9922-7 29995244

[29] Maya RM, Nona S. Assessment of rotahaler inhalation technique among patients with COPD or asthma at Manipal teaching hospital, Pokhara. Int J Nurs Educ. 2018;10: 91. doi: 10.5958/0974-9357.2018.00074.0

[30] van Beusekom MM, Grootens-Wiegers P, Bos MJW, Guchelaar HJ, van den Broek JM. Low literacy and written drug information: information-seeking, leaflet evaluation and preferences, and roles for images. Int J Clin Pharm. 2016;38: 1372–1379. doi: 10.1007/s11096-016-0376-4 27655308 PMC5124048

[31] Branin JJ. The Role of Memory Strategies in Medication Adherence Among the Elderly. Home Health Care Serv Q. 2001;20: 1–16. doi: 10.1300/J027v20n02_01 11987652

[32] Dijkstra NE, Sino CGM, Schuurmans MJ, Schoonhoven L, Heerdink ER. Medication self-management: Considerations and decisions by older people living at home. Res Soc Adm Pharm. 2022;18: 2410–2423. doi: 10.1016/j.sapharm.2020.09.004 33627223

[33] Jin HK, Kim YH, Rhie SJ. Factors affecting medication adherence in elderly people. Patient Prefer Adherence. 2016;10: 2117–2125. doi: 10.2147/PPA.S118121 27799748 PMC5077271

[34] Barros IMC, Alcântara TS, Mesquita AR, Bispo ML, Rocha CE, Moreira VP, et al. Understanding of pictograms from the United States pharmacopeia dispensing information (USP-DI) among elderly Brazilians. Patient Prefer Adherence. 2014;8: 1493–1501. doi: 10.2147/PPA.S65301 25378914 PMC4219639

[35] Monteiro S P., van Weert J C.M., de Gier J J., van Dijk L. Age and education related preferences for pictograms concerning driving-impairing medicines. Heal Prim Care. 2017;1: 1–6. doi: 10.15761/hpc.1000107

[36] Santos-Pérez MI, Fierro I, Salgueiro-Vázquez ME, Del Mar Gallardo-Lavado M, Sáinz-Gil M, Martín-Arias LH. A polypharmacy risk prediction model for elderly patients based on sociodemographic and clinical factors. Int J Clin Pharmacol Ther. 2018;56: 577–584. doi: 10.5414/CP203238 30336804

[37] Benjamin RM. Multiple Chronic Conditions: A Public Health Challenge. Public Health Rep. 2010;125: 626–627. doi: 10.1177/003335491012500502 20873276 PMC2924996

[38] Voigt K, Gottschall M, Köberlein-Neu J, Schübel J, Quint N, Bergmann A. Why do family doctors prescribe potentially inappropriate medication to elderly patients? BMC Fam Pr. 2016;17. doi: 10.1186/s12875-016-0482-3 27449802 PMC4957869

[39] Clyne B, Cooper JA, Boland F, Hughes CM, Fahey T, Smith SM. Beliefs about prescribed medication among older patients with polypharmacy: A mixed methods study in primary care. Br J Gen Pract. 2017;67: e507–e518. doi: 10.3399/bjgp17X691073 28533200 PMC5540192

[40] Park HY, Seo SA, Yoo H, Lee K. Medication adherence and beliefs about medication in elderly patients living alone with chronic diseases. Patient Prefer Adherence. 2018;12: 175–181. doi: 10.2147/PPA.S151263 29416319 PMC5790098

[41] Bultman DC, Svarstad BL. Effects of physician communication style on client medication beliefs and adherence with antidepressant treatment. Patient Educ Couns. 2000;40: 173–185. doi: 10.1016/s0738-3991(99)00083-x 10771371

[42] Droppert H, Bennett S, Singh J, Singh N, Kumar R, Bhandari V, et al. Awareness about prescribed drugs among patients attending Out-patient departments. Int J Appl Basic Med Res. 2013;3: 48. doi: 10.4103/2229-516X.112240 23776839 PMC3678681

