# Supplementary material for: Self-administration medication errors at home and its predictors among illiterate and low-literate community-dwelling older adults with polypharmacy: A negative binomial hierarchical regression
Source: PLoS One. 2024 Apr 19;19(4):e0302177. doi: 10.1371/journal.pone.0302177 (PMC11029665; doi:10.1371/journal.pone.0302177)
Supplement: S1 File — (DOCX) [file pone.0302177.s001.docx]

**Appendix 1: Checklist of medication self-administration errors (MSEs) in the older adults**

| **Any unintentional discrepancy between the medicine prescription and the participant’s answers is considered a medication error (not taking medicine as prescribed by a doctor or instructed by a pharmacist)** | | | | |
| --- | --- | --- | --- | --- |
| **Type of error** | | **Definition and examples of error** | **Yes** | **No** |
| *Forgetting to take medicines* | | Failure to take prescribed medicine. |  |  |
| *Improper timing* | | Taking medicine at a time other than prescribed by a physician, especially when its effectiveness is affected. For example, taking hypnotics in the morning, or taking medications at an improper time. |  |  |
| *Incorrect dosage* | *Higher dose* | Taking a higher dosage of medicine prescribed by the physician. For example, the doctor said to take half of a tablet, and the patient takes the whole tablet; or taking extra units of insulin; or using a tablespoon instead of a teaspoon for taking syrups; or using more drops of eye drops. |  |  |
|  | *Lower dose* | Taking a lower dosage of medicine prescribed by the physician. |  |  |
|  | *Duplication* | Repeated taking of the same medicine. |  |  |
| *Taking the wrong medication because of similar appearance (look-alike packaging)*  *The term LASA (“lookalike sound-alike”) delineates a confusion of medication due to the similar labeling and packaging of different*  *drugs, or similar labeling and packaging of the same drug containing different strengths.* | | Taking the wrong medicine instead of a medication prescribed by a physician because of their similar appearance. For example, confusing eardrops and eyedrops |  |  |
| *Incorrect route of administration* | | The wrong way to take the medicine in a way that causes serious, sometimes long-term side effects or reduces medication efficacy. For example, swallowing suppositories or Spiriva; swallowing chewable medicines, or splitting Entric Coat (E.C) tablets |  |  |
| *Improper taking of medications with food or, without food beverages, and herbs* | | The medications have major interaction with food, beverages, and herbs for example, the patient should not have food, drink, medications, or supplements for at least one half-hour after taking Bisphosphonates, Ciprofloxacin, Norfloxacin, Tetracycline, Azithromycin, and Levothyroxine, Captopril, Omeprazole, Pantoprazole.  Medicines that cause side effects if not taken with food and should be taken with meals. For example, taking Prednisone or NSAIDs on an empty stomach.  Medications with special considerations for example using foods or herbal products that could affect medication absorption. For example, using foods containing high vitamin K (green leafy vegetables such as kale, collard greens, broccoli, spinach, cabbage, and lettuce.) with warfarin or using high tyramine foods with Monoamine Oxidase Inhibitors (MAOI). |  |  |
| *Improper taking of medications together* | | Medications with major drug-drug interactions which physician emphasized not to use together. For example, simultaneous taking of Warfarin and Macrolides. |  |  |
| *Medication omission* | | The unintentional omission of one or more drugs from the prescribed treatment regimen. For example, taking losartan instead of losartan H. |  |  |
| *Taking previous medications* | | Continued taking of medications prescribed by previous doctors and/or hospitals without knowing whether they are needed or not. |  |  |
| *Taking another person's medications* | | Take another person's or pet's medicine by mistake. Keeping medications next to each other on the table. |  |  |
| *Errors related to medication delivery involve devices* | | Misuse of medication devices such as insulin pens or inhalers. For example, failure to follow device's instructions correctly. |  |  |
| *Improper Medication Storage* | | Incorrect storage of medications results in the destruction of medicines and affect the quality of them. For example, keeping the medicine in a warm place (near the heater), exposed to sunlight, or Keeping medicine in a part of the refrigerator that causes freezing. |  |  |
| *Taking expired medications* | | Unintentional use of expired medicine. |  |  |
| *Self-medication (taking medications without a prescription)* | | Chronic use of medicines that are taken without a doctor's prescription. Short-term or occasional use of OTC medications is not classified as self-medication. |  |  |
| *Forgetting the doctor´s or pharmacist´s instructions* | | Uncertainty about the instructions given by the doctor or pharmacist |  |  |
| *Mistaken drug because of misunderstanding of the purpose of the medication* | | For example, taking Mefenamic acid for stomach pain. |  |  |
| *Other medication-related errors* | | Any error that is related to the use of medication but does not fall under the category of other errors. For example, swallowing silica gel |  |  |
| The examples listed in the table for each error were part of the examples of medication errors.  Drug-drug and drug-food interactions and other supplementary investigations were done by the pharmacist using medication review. | | | | |
